# Free-living nematode assemblages associated with maize residues and their ecological significance

**DOI:** 10.21307/jofnem-2021-038

**Published:** 2021-04-01

**Authors:** Samuel Maina, Hannah Karuri, Rossa Nyoike Ng’endo

**Affiliations:** Department of Biological Sciences, University of Embu, P.O. Box 6-60100, Embu, Kenya

**Keywords:** Decomposition, Ecological significance, Free-living nematodes, Maize residue, Soil food web

## Abstract

Return of plant residues to the soil is a sustainable way of enhancing plant growth, health, and levels of soil quality. In Kenya, maize plant residues are the most commonly returned plant material in many agro-ecosystems. For any plant material to release nutrients into the soil, it must undergo a decomposition process that is usually affected by various organisms, especially nematodes. Despite their great contribution to the breakdown of plant organic matter, there is a dearth of information on the interaction between maize residues and free-living nematodes (FLN) in Kenya. In this respect, this study aimed to assess the influence of decomposing maize residues on FLN dynamics and the soil food web in Mwea, Kenya. The experimental plots were set up in a randomized complete block design, comprising of decomposition plots (incorporated with maize residue to a depth of 30 cm at a rate of 5 tons/hectare), while the plots unincorporated with maize residues were used as the control. Each treatment consisted of four replicates. In all, 30 FLN genera were recovered from the field trials, whereby *Acrobeles* was significantly abundant in decomposition plots in both seasons. We subsequently found that maize residues reduced the abundance of enrichment opportunist bacterivores (cp-1) relative to general opportunist (cp-2) bacterivores and fungivores. Notably, the results of the channel index showed that the decomposition of maize residues was dominated by fungal energy channels throughout the study in the two seasons. These results suggest that maize residues need to be coupled with a suitable labile organic matter. This would lead to sustainable, active, and reliable turn-over of maize residues into the soil food web ecosystems. The application of labile materials can also help to improve the population of enrichment bacterivores that are essential in the decomposition process. This study shows that the decomposition of maize residues influenced FLN composition, mainly the enrichment opportunist bacterivores whose abundance was lower.

Decomposition is a microbial process that involves the breakdown of complex organic compounds into less complex inorganic materials (De Mesel et al., 2006; Yadav et al., 2018). This process is fundamental for the release of immobilized nutrients such as nitrogen from plant residues into the soil. Plant residues, including maize leaves and stalks, provide energy to the soil food web in form of organic carbon and nitrogen, and also protect the soil against rain and wind erosion. In addition, plant residues are composed of complex compounds primarily lignin, cellulose, and hemicellulose. Conversely, microbes have been found to breakdown these compounds into nutrients forms that are easily available for plant uptake (Chaves et al., 2004). The decomposition rate is usually influenced by environmental factors including moisture and temperature, C to N ratios in the plant residues, and the number/type of organisms and their interactions (Bengtsson et al., 1995; Ha et al., 2008). More importantly, the C to N ratio of plant residues has been shown to exhibit a significant effect on decomposition (USDA NRCS, 2011). For example, if wheat straw (C to N = 80:1), hairy vetch (C to N = 11:1), and alfalfa hay (C to N = 25:1) are incorporated into the soil, the vetch and alfalfa will be decomposed more rapidly than wheat straw. Likewise, young alfalfa hay (C to N = 13:1) will also decompose more quickly compared with rye straw (C to N = 82:1) and corn stover (C to N = 57:1) due to lower C to N ratio.

Free-living nematodes (FLN) also hold an integral position in decomposition (Neher, 2010; Yadav et al., 2018). These nematodes closely interact with soil microbes and contribute either directly or indirectly to N mineralization through accelerated turnover of microbial cells, modification of the microbial community, and addition of new substrates, thereby increasing the availability of N (Mekonen et al., 2017; Sultana and Bohra, 2012; Yadav et al., 2018). In the soil food web, the decomposition of organic matter can be divided into two energy channels, including a faster bacterial facilitated channel and a slower fungal mediated channel (Ferris et al., 2001). These two channels run simultaneously, and they are dynamic features in the soil ecosystem (Ruess and Ferris, 2004). The prevalence of a specific decomposition channel in the food web is primarily determined by the type of soil and nutrient forms of organic plant residues (Ferris and Matute, 2003; Ingham et al., 1985). According to Ingham et al. (1985), bacteria and fungi play a pivotal role in the soil food web decomposition, particularly mineralizing nutrients from forms otherwise unavailable for plant uptake.

Monitoring consumer organisms of bacteria and fungi (bacterivores and fungivores, respectively), as well as other nematode assemblages such as omnivores and predators, may serve as good indicators of changes that occur in the soil (Ruess and Ferris, 2004). For instance, it has been demonstrated that overgrazing of bacteria and fungi by nematodes (bacterivores and fungivores, respectively) can lower the overall activity of these decomposers (Ferris et al., 2012a). Interestingly, the soil food web structure consists of higher trophic groups (omnivores and predators), which exert a regulatory function on bacterivores and fungivores, thus increasing the cycling of nutrients (Ferris et al., 2012b; Yeates and Wardle, 1996). Neher (2001) observed that nematode excretion may provide approximately 19% of soluble nitrogen into the soil. Under field conditions, studies have estimated that bacterivores and predators contribute, directly or indirectly, around 8 to 19% of N mineralization in conventional and integrated farming systems, respectively (Beare, 1997; Neher, 2010).

Moreover, multiple reports have established that the contribution of nematodes to N mineralization is relatively higher than that of bacteria, and besides N mineralization, they are also capable of mineralizing many other soil nutrients, such as phosphorous, thus enhancing plant growth (Gebremikael et al., 2016; Pokharel, 2011; Wang et al., 2004). The purpose of this study was to assess the influence of decomposing maize residues on the abundance and diversity of free-living nematodes. In this context, we also evaluated the ecological role of these nematodes.

## Materials and methods

### Site description

This study was carried out in two separate trials (March–July 2018 and October–February 2019) at Nyangati, Mwea Sub-county, Kenya (Longitude 0°36'45.5“S and Latitude 37°21'18.0“E). The region is classified in agroecosystem zone 3 at 1,202 m in lowland altitude. The soil type in the area is characterized as vertisols (Jaetzold et al., 2009), and receives two rainy seasons per year. The long rains (from March to May) average about 900 mm, whereas short rains (from October to November) average around 800 mm. The minimum temperature and average rainfall recorded in the study area are provided in Table 1, while soil physicochemical properties are given in Table 2.

**Table 1.** Minimum temperature and rainfall data at Nyangati study site in Mwea Sub-county, Kenya.

Month	Minimum temperature (°C)	Average rainfall (mm)
March	14.6	245
April	14.7	619.2
May	14.8	299.6
June	13.3	78
July	12.7	51.5
August	12.3	18.8
September	13.0	38.5
October	14.6	51.5
November	15.5	100.1
December	14.7	109.2
January	13.6	46.5
February	21.6	21.6

**Table 2.** Soil physicochemical characteristics at Nyangati study site in Mwea Sub-county, Kenya.

Soil property	Mean	Standard error
Calcium me %	6.07	0.788
Clay %	44.00	4.000
Copper ppm	2.95	0.090
Iron ppm	23.50	3.239
Magnesium me %	3.63	0.135
Manganese me %	0.97	0.114
Phosphorus ppm	16.67	1.667
Potassium me %	0.75	0.096
Sand %	46.67	3.712
Silt %	9.33	0.667
Sodium me %	0.16	0.074
Soil pH	5.85	0.057
Total Nitrogen %	0.18	0.003
Total Organic Carbon %	2.03	0.050
Zinc ppm	0.20	0.029

Note: me = milliequivalent.

### Field experimental design

This work comprised of two experimental groups, consisting of decomposition plots (incorporated with maize residues at the rate of 5 tons/hectare, which is the rate commonly used by many Kenyan farmers) and control plots, where no maize residues were incorporated. The field plots were arranged in a randomized complete block design (RCBD), with each experimental group consisting of four replicates. Each plot measured 36 m^2^ (6 × 6 m) and was separated by a 1 m buffer zone between plots. In addition, each plot had six rows per plot. The maize residues were first chopped into small pieces (approximately 10 cm) to enhance the decomposition process and subsequently incorporated into the soil to a depth of 30 cm.

### Soil sampling and design

Soil samples were taken monthly for a period of 4 months after the incorporation of maize residue for two seasons (March–July 2018 and October–February 2019). In each plot, five soil cores (100 g each) were randomly collected from the inner rows at 0–20 cm depth, using a soil auger measuring 3.5 cm in diameter through a cross diagonal sampling pattern. During each sampling date, the five soil sub-samples were mixed gently and composited into a 500 g homogenous sample (Coyne et al., 2014).

### Nematode extraction, enumeration, and classification

Each soil sample (250 g derived from a 500 g composite sample) was placed in filter trays and nematodes were extracted and recovered for 48 h using a modified Baermann technique (Hooper et al., 2005). After extraction, nematodes were enumerated by counting and identified to genus level under a compound microscope using diagnostic keys. Afterwards, the FLN nematode genera were classified into four functional guilds, including bacterivores, predators, fungivores, and omnivores (Yeates et al., 1993). They were also assigned into their respective colonizer-persister group, from cp-1 to cp-5, where index values represent life course strategies that are attributed with either r- or K- characteristics (Ferris et al., 2001). Those nematodes assigned to cp-1 are referred to as colonizers that are usually characterized by high fecundity and small eggs. They also require enriched nutrients for favorable growth, have large population changes, and short generation times. Those assigned to cp-5 are known as persisters, which are characterized by few offspring, low fecundity, large body size, and great sensitivity to disturbances (Bongers and Bongers, 1998).

### Weather and soil physicochemical characteristics data

Data on average rainfall and minimum temperature (Table 1) during the field trials were obtained from Kenya Meteorological Department, Nairobi, Kenya. The soil physicochemical properties (calcium, clay, copper, iron, magnesium, manganese, phosphorus, potassium, sand, silt, sodium, pH, nitrogen, total organic carbon, and zinc) were analyzed at Kenya Agricultural and Livestock Research Organization, Nairobi, Kenya (Table 2).

### Data analysis

Prior to the analysis, log transformation (Log (*x* + 1)) of nematode count data was done where necessary to meet the normality criteria for statistical data analyses. The influence of maize residues on nematode abundance, ecological, functional, and metabolic footprint indicators was assessed using ANOVA with the R package vegan (Oksanen et al., 2016). The treatment means separation was implemented with Tukey HSD test using the R package agricolae (De Mendiburu, 2015). We evaluated the ecological significance of FLN using ecological, functional, and metabolic footprint indicators. In particular, the ecological indices (Maturity indices; MI and MI2-5) were computed to infer soil food web ecosystem perturbations (Ferris and Bongers, 2009). Then, the functional soil food web indices, including channel index (CI), structure index (SI), basal index (BI), and enrichment index (EI) were evaluated to infer the status and complexity of the soil food web conditions as indicated by nematode assemblage composition (Ferris et al., 2001). Nematode metabolic footprints were explored to indicate various ecosystem functions, services, and structure by partitioning the amount of carbon utilized by nematodes in metabolic activity (respiration) and growth and egg production (biomass) (Ferris, 2010). All ecological, functional, and metabolic footprint indicators were computed using the online program, Nematode Indicator Joint Analysis (Sieriebriennikov et al., 2014). All statistical analysis was performed using R software, version 3.6.2 (R Core Development Team, 2019).

## Results

### Nematode composition and abundance

Thirty free-living nematode genera assigned to four trophic levels were identified. The most predominant trophic guild in all decomposition plots in both seasons was bacterivores, followed by fungivores. We observed significant treatment effects (*P* < 0.05) on a total of 8 nematode genera (first season) and 13 genera (second season) (Tables 3 and  4, respectively). In the first season, there were 15 bacterivores, 4 fungivores, 3 omnivores, and 3 predators. Among the different taxa, *Acrobeles* and *Plectus* (bacterivores), *Aporcelaimellus* (omnivore), and *Nygolaimus* and *Seinura* (predators) were the most abundant genera in the decomposition plots (Table 3). On the other hand, in the second season, there were 15, 3, 6, and 3 bacterivores, fungivores, omnivores, and predators, respectively. Among the various taxa, *Cephalobus*, *Eucephalobus*, *Acrobeles, Heterocephalobus*, *Aphelenchus,*  and *Aphelenchoides* were significantly higher in decomposition treatments, while *Mesodorylaimus* (omnivore), *Nygolaimus* and *Mononchus* (predators) were more prevalent in the control plots (Table 4). Most of the bacterivores were classified as cp-1 (Ba1) and cp-2 (Ba2) except *Alaimus* and *Prismatolaimus*, while all fungivores were assigned to cp-2 (Fu2). Both omnivores and predators were categorized as cp-4 and cp-5 except for *Seinura*. We further identified that the abundance of cp-1 bacterivores was lower in the decomposition plots than in the control plots, but this was not the case for generalist opportunist (cp-2) bacterivores and fungivores.

**Table 3.** Average number of nematodes in 250 g soil collected from control and decomposition plots during season one.

Taxa	Cp-class	Control Mean ± SE	Decomposition Mean ± SE	*F*-value	*P*-value
***Bacterivores***					
*Acrobeles*	2	42 ± 9.5	58 ± 8.0	37.039	<0.001***
*Acrobeloides*	2	16 ± 4.4	8 ± 3.5	9.153	0.005**
*Alaimus*	4	31 ± 8.8	30 ± 5.7	2.331	0.137
*Aphanolaimus*	1	5 ± 2.4	2 ± 1.1	1.327	0.258
*Cephalobus*	2	107 ± 6.4	109 ± 9.9	0.106	0.747
*Cervidellus*	2	52 ± 11.3	23 ± 3.8	0.082	0.777
*Drilocephalobus*	2	0 ± 0.0	3 ± 2.0	1.946	0.173
*Eucephalobus*	2	91 ± 12.9	118 ± 21.1	2.92	0.098
*Geomonhystera*	2	10 ± 4.8	7 ± 3.6	0	0.996
*Heterocephalobus*	2	120 ± 20.9	86 ± 13.4	1.927	0.175
*Plectus*	2	26 ± 9.0	37 ± 7.6	7.016	0.013*
*Prismatolaimus*	3	18 ± 4.0	40 ± 11.0	2.586	0.118
*Rhabditis*	1	39 ± 8.0	41 ± 12.6	0.223	0.64
*Stegelleta*	2	0 ± 0.0	1 ± 1.3	0.272	0.325
*Wilsonema*	2	31 ± 10.1	17 ± 4.2	0.679	0.417
***Fungivores***					
*Aphelenchoides*	2	16 ± 5.8	9 ± 3.2	1.088	0.305
*Aphelenchus*	2	143 ± 1.0	181 ± 19.0	2.479	0.126
*Ditylenchus*	2	13 ± 4.6	1 ± 1.3	49.301	<0.001***
*Filenchus*	2	16 ± 2.2	9 ± 2.7	9.997	0.004**
***Omnivores***					
*Aporcelaimellus*	5	0 ± 0.0	4 ± 1.9	4.719	0.038*
*Eudorylaimus*	4	8 ± 2.4	3 ± 1.3	3.684	0.064
*Labronema*	4	62 ± 4.0	65 ± 8.0	1.196	0.283
***Predators***					
*Discolaimus*	5	8 ± 2.4	10 ± 3.3	0.076	0.784
*Nygolaimus*	5	36 ± 6.9	42 ± 6.4	9.345	0.005**
*Seinura*	2	0 ± 0.0	6 ± 2.4	22.167	<0.001***

Notes: Cp = colonizer-persister value. *P < 0.05; **P < 0.01; ***P < 0.001.

**Table 4.** Average number of nematodes in 250 g soil collected from control and decomposition plots during season two.

Taxa	Cp*-*class	Control Mean ± SE	Decomposition Mean ± SE	*F*-value	*P*-value
***Bacterivores***					
*Acrobeles*	2	39 ± 7.5	58 ± 12.8	6.604	0.015*
*Acrobeloides*	2	42 ± 8.0	27 ± 6.0	1.434	0.241
*Alaimus*	4	8 ± 2.4	10 ± 3.9	0.059	0.81
*Cephalobus*	2	151 ± 10.4	280 ± 26.9	25.737	<0.001***
*Cervidellus*	2	42 ± 13.5	34 ± 12.1	0.466	0.5
*Drilocephalobus*	2	5 ± 2.4	9 ± 3.2	4.225	0.049*
*Eucephalobus*	2	101 ± 19.1	164 ± 23.8	48.172	<0.001***
*Geomonhystera*	2	16 ± 3.5	8 ± 3.0	7.391	0.0108*
*Heterocephalobus*	2	57 ± 10.8	129 ± 14.8	68.576	<0.001***
*Mesorhabditis*	1	0 ± 0.0	14 ± 4.9	37.891	<0.001***
*Panagrolaimus*	1	3 ± 1.2	5 ± 2.0	0.486	0.491
*Plectus*	2	10 ± 3.5	27 ± 4.7	96.111	<0.001***
*Prismatolaimus*	3	16 ± 3.5	24 ± 4.2	3.989	0.055
*Rhabditis*	1	21 ± 7.0	73 ± 34.4	2.592	0.118
*Wilsonema*	2	3 ± 1.2	3 ± 1.5	0.207	0.652
***Fungivores***					
*Aphelenchoides*	2	29 ± 6.1	98 ± 29.5	13.911	<0.001***
*Aphelenchus*	2	164 ± 20.9	320 ± 47.2	18.84	<0.001***
*Filenchus*	2	21 ± 4.8	16 ± 6.0	2.041	0.163
***Omnivores***					
*Aporcelaimellus*	5	0 ± 0.0	1 ± 1.3	1	0.325
*Eudorylaimus*	4	26 ± 4.2	22 ± 5.2	1.34	0.256
*Dorylaimellus*	5	0 ± 0.0	1 ± 0.7	1	0.325
*Labronema*	4	39 ± 7.1	47 ± 7.1	2.561	0.12
*Mesodorylaimus*	4	23 ± 3.5	10 ± 2.9	13.006	0.001**
*Prodorylaimus*	4	0 ± 0.0	3 ± 1.5	3.649	0.066
***Predators***					
*Discolaimus*	5	8 ± 1.5	8 ± 2.0	0.051	0.823
*Mononchus*	4	5 ± 1.5	4 ± 2.7	4.741	0.037*
*Nygolaimus*	5	78 ± 10.3	54 ± 9.9	5.483	0.026*

.

### Ecological, functional, and nematode metabolic footprint indicators

**Figure 1:**
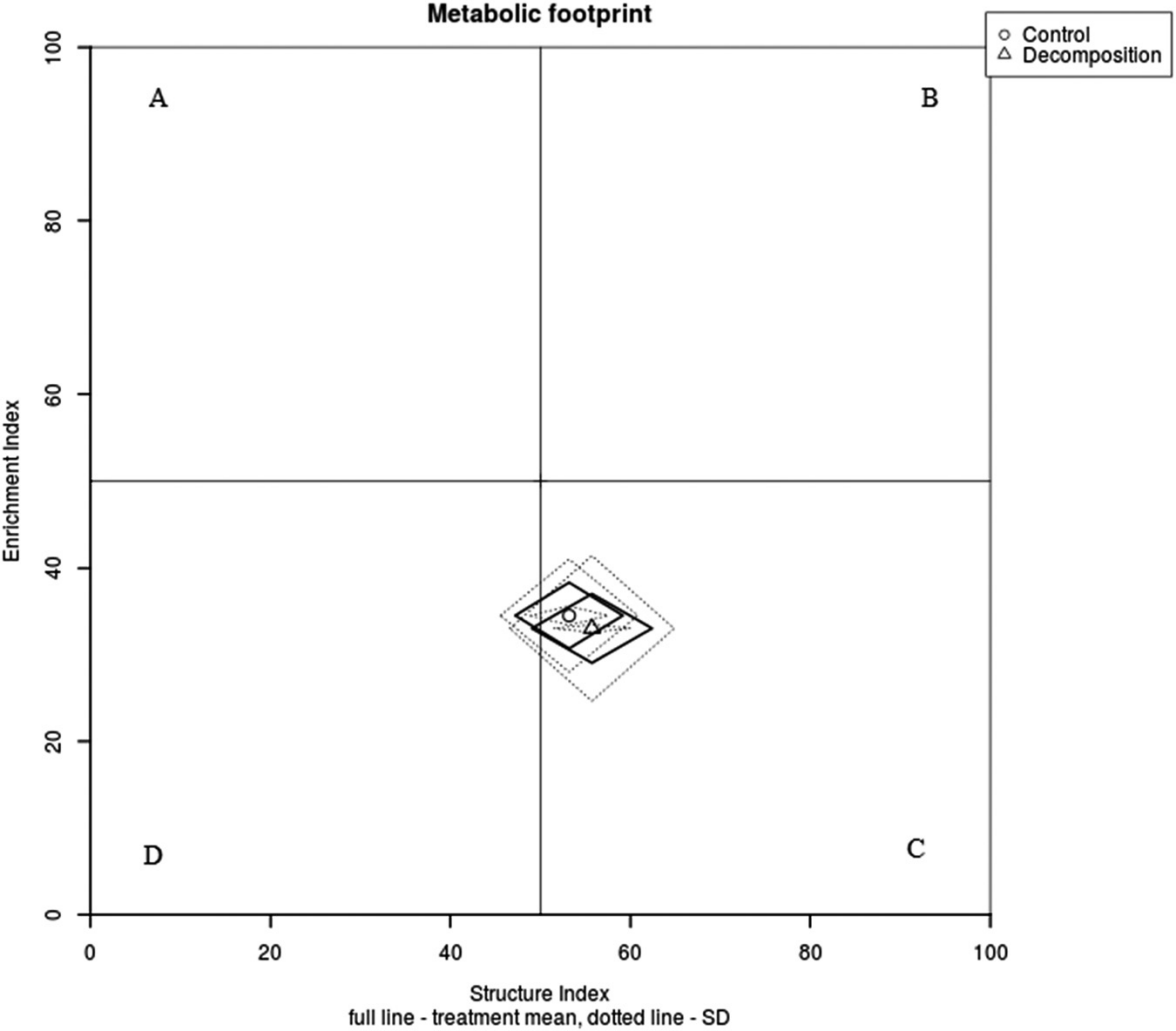
Functional metabolic footprint of nematodes in control and decomposition plots during the first season. The point at the middle of the rhomboid represents the intersection of the enrichment and structure index. The length of the vertical axis and horizontal axis of the rhombus represents the enrichment and structure footprints, respectively. The functional nematode metabolic footprint is represented by the total area of the functional enrichment and structure footprints.

**Figure 2:**
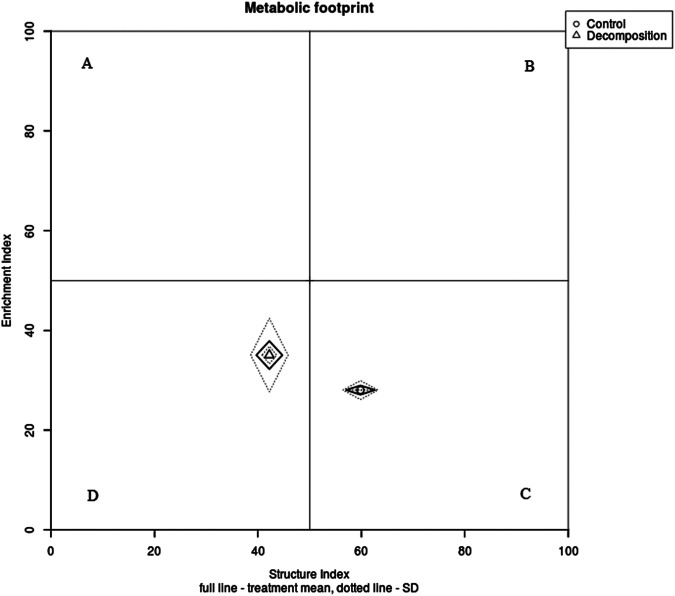
Functional metabolic footprint of nematodes in control and decomposition plots during the second season. The point at the middle of the rhomboid represents the intersection of the enrichment and structure index. The length of the vertical axis and horizontal axis of the rhombus represents the enrichment and structure footprints, respectively. The functional nematode metabolic footprint is represented by the total area of functional enrichment and structure footprints.

There were no differences in ecological indicators (MI, MI2-5) and functional indicators (CI, BI, EI, and SI) in season one at *P* < 0.05 (Table 5). However, during the second season, all the values of MI, MI2-5, CI, BI, EI, and SI were significantly affected at *P* < 0.001 (Table 5). In particular, maturity indices (MI and MI2-5), CI, and SI were reduced in the decomposition plots. Conversely, BI and EI were markedly increased at *P* < 0.001 in the decomposition plots relative to the control. Moreover, the metabolic footprints (composite, structure, predator, fungivore, enrichment, and bacterivore) were statistically different (*P* < 0.05) during the second season (Table 6). A combination of fungivore, enrichment, and bacterivore footprints, an indicator for mineralization services in the soil food web, was significantly larger in the decomposition treatments than in the control plots in the same season. We then observed that structure and predator footprints developed a lower activity in the decomposition plots in the second season, while the composite footprint was progressively higher in the decomposition plots compared with the control in both seasons (Table 6). Notably, in season one, only predator footprint was statistically different between decomposition and control plots (*P* < 0.001) (Table 6). Finally, plotting nematode functional metabolic footprints using EI and SI revealed that both control and decomposition plots were characterized as structured (quadrat “C”) in season one (Figure 1). During the second season, the control remained structured (quadrat “C”), while the decomposition plots shifted to quadrat “D” categorized as degraded (Figure 2).

**Table 5.** Nematode ecological and functional indices in control and decomposition plots during season 1 and 2.

	Control	Decomposition		
Index name	Mean ± SE	Mean ± SE	*F*-values	*P*-value
*Season 1*				
Maturity Index (MI)	2.41a ± 0.038	2.43a ± 0.040	0.38	0.542
Maturity Index (MI2-5)	2.47a ± 0.044	2.49a ± 0.036	0.409	0.528
Channel Index (CI)	60.35a ± 5.95	65.66a ± 5.948	0.985	0.329
Basal Index (BI)	37.82a ± 2.016	35.72a ± 1.652	1.452	0.238
Enrichment Index (EI)	34.52a ± 1.455	33.03a ± 2.472	0.511	0.481
Structure Index (SI)	53.19a ± 2.537	55.73a ± 2.224	1.435	0.24
*Season 2*				
Maturity Index (MI)	2.54a ± 0.033	2.26b ± 0.036	64.715	<0.001***
Maturity Index (MI2-5)	2.57a ± 0.031	2.33b ± 0.029	68.996	<0.001***
Channel Index (CI)	81.01a ± 4.923	68.37b ± 5.864	12.359	<0.001***
Basal Index (BI)	34.44b ± 1.267	42.47a ± 1.495	65.944	<0.001***
Enrichment Index (EI)	28.14b ± 2.519	35.06a ± 2.941	15.409	<0.001***
Structure Index (SI)	59.91a ± 1.535	42.21b ± 2.689	41.205	<0.001***

**Table 6.** Metabolic footprints in control and decomposition plots during season 1 and 2.

	Control	Decomposition		
Metabolic footprints	Mean ± SE	Mean ± SE	F-values	*P*-value
*Season 1*				
Composite footprint	5.67a ± 3.444	5.77a ± 3.421	2.482	0.126
Omnivore footprint	4.55a ± 1.750	4.35a ± 2.558	0.825	0.371
Structure footprint	4.99a ± 2.345	5.07a ± 2.702	1.272	0.268
Predator footprint	3.15b ± 2.127	3.82a ± 2.110	19.678	<0.001***
Fungivore footprint	2.91a ± 1.243	2.94a ± 1.101	0.126	0.725
Enrichment footprint	4.326a ± 2.797	4.196a ± 3.262	0.556	0.462
Bacterivore footprint	4.734a ± 3.267	4.918a ± 3.303	2.441	0.129
*Season 2*				
Composite footprint	5.68b ± 3.242	5.95a ± 4.301	11.018	0.002**
Omnivore footprint	4.23a ± 2.479	4.33a ± 2.657	0.679	0.417
Structure footprint	5.22a ± 2.314	5.05b ± 2.870	4.500	0.042*
Predator footprint	4.48a ± 2.485	3.87b ± 2.515	5.894	0.021*
Fungivore footprint	2.89b ± 1.260	3.52a ± 1.972	21.683	<0.001***
Enrichment footprint	3.51b ± 2.755	4.43a ± 4.249	27.425	<0.001***
Bacterivore footprint	4.39b ± 2.945	5.10a ± 4.269	30.782	<0.001***

## Discussion

Decomposition is a valuable process for restoring energy fluctuations in terrestrial environments. Recycling plant residues accompanied by the breakdown of these materials into nutrients by belowground decomposer systems enhances plant life and levels of soil fertility (Ruess and Ferris, 2004). This study showed seasonal variations in the population of bacterivores, omnivores, fungivores, and predators across all treatments. The most abundant bacterivore genera in the decomposition plots compared to the control belonged to the family Cephalobidae (*Acrobeles*; season one and *Cephalobus*, *Eucephalobus*, *Heterocephalobus,*  and *Acrobeles*; season two), and Plectidae (*Plectus*; season one). These bacterivores are characterized by high reproduction rates, short life cycles, and persist in both poor and enriched food conditions (Bongers and Bongers, 1998; Yeates, 2003). In both seasons, the cp-2 bacterivores, which are classified as general opportunists, were more prevalent than cp-1 bacterivores, categorized as enrichment opportunists, in the decomposition plots. These observations may be attributed to the fact that maize residues, being a recalcitrant material, probably favored the higher abundance of cp-2 bacterivores than that of cp-1 bacterivores, which were fewer. This is in agreement with Ferris and Matute (2003), who noted fewer cp-1 bacterivores with more recalcitrant material with high C to N ratios.

The fungivorous taxa *Aphelenchus* and *Aphelenchoides,* belonging to the cp-2 functional guild that is classified as general opportunists, were the most dominant fungal feeding decomposers in the decomposition plots in season two, while in season one, there were no significant differences between control and decomposition plots. Increased abundance of these fungivore opportunists may be explained by the possibility that the input of maize residues perhaps enhanced conducive conditions by providing necessary energy resources for fungal activity. This might have increased the fungal population, which the fungivores fed on, subsequently increasing their population density (Ferris et al., 2012c; Wasilewska et al., 1981). Neher (2010) also reported an increase in fungivorous nematodes in relation to fungi during the decomposition of plant organic compounds that was ascribed to a high lignin content to nitrogen ratio of the plant material. The most dominant omnivore and predator genera in decomposing maize residue plots were *Aporcelaimellus* and *Nygolaimus* (season one). The increase in abundance of these nematodes belonging to the higher trophic guild (cp-4 and cp-5) may be essential for soil health, because these nematodes hold a significant function as system regulators of the activity of both bacterial and fungal feeding nematodes (Ferris et al., 2012b; Yeates and Wardle, 1996), thus improving nutrient mineralization in the food web. Overgrazing of fungi and bacteria populations by bacterivores and fungivores can affect their activity and in turn interfere with the decomposition process (Yadav et al., 2018). During the second season, *Mesodorylaimus*, *Nygolaimus,* and *Mononchus* were progressively more abundant in control compared to decomposition plots. This implies that the lower dominance of these nematodes in the decomposition plots could be associated with changes in climatic conditions or higher predation pressure from other organisms such as tardigrades.

The Channel index (CI), reflects the nature of the decomposition of organic matter either through fungal or bacterial energy channels (Ferris et al., 2001). Lower CI values < 50% reflect bacterial energy pathways while CI > 50% indicate fungal decomposition activity (Ferris et al., 2001). For both seasons, findings from this study depict CI values that range between ~66 and ~68 in all decomposition plots. This designates the dominance of fungal decomposition activity in decomposing maize residues. The dominance of fungal energy channel may have been favored by maize residues perhaps due to its high C to N ratio, and also because fungi are able to degrade more complex polyaromatic compounds such as cellulose and lignin, thus providing more food resources to fungivores (Rosenbrock et al., 1995; Ruess and Ferris, 2004).

Maturity indices, (MI) and (MI2-5), are indicators of structural complexity and conditions of a succession of soil food web environments as reflected by various nematode community assemblages (Ferris and Bongers, 2009). Our results revealed that the values of MI and MI2-5 in both decomposition and control plots ranged between 2 and 3.5. This implies low to intermediate soil food web maturity, balanced organic matter decomposition, and medium soil food web structure in both seasons. A relatively high population of fungivores increased the level of the basal index (BI) in decomposition plots (season two), while in season one, it was not significant (*P* = 0.238). The basal index was primarily developed to detect soil food web perturbations through nematode trophic groups that are tolerant to various disturbances (Ferris et al., 2001). In this study, BI was more sensitive to decomposing maize residues. In contrast, Li et al. (2010) noted that BI was reduced by organic amendments relative to the control. Ferris et al. (2001) suggested that coupling EI and CI provides an interesting basis for evaluating the levels of active organic matter decomposition. In the present study, significantly higher values of EI and lower CI in decomposition treatments than in the control during the second season implies more enriched soil food web conditions with greater fungal activity. Similar findings were observed by Sánchez-Moreno et al. (2010) and Zhang et al. (2012).

Structure index (SI) was designed to estimate the complexity of the soil food web structure based on higher trophic levels (cp-3, cp-4, and cp-5) (Ferris et al., 2001). Our findings demonstrated that SI was significantly (*P* < 0.001) lower in decomposition plots during the second season, classifying soil food web conditions as degraded. Similarly, this was also reflected in the functional metabolic footprint profile analysis in the same season. In addition, higher levels of fungivore, enrichment, and bacterivore footprints (an indicator for mineralization services) were considerably higher in decomposition plots (second season), whereas in the first season, they were similar between decomposition and control plots. This implies that recycling of maize residues may provide necessary mineralization services that are desired conditions for good agro-ecosystems (Sánchez-Moreno and Ferris, 2018). Ferris (2010) established that a higher composite footprint indicates that the whole nematode community composition accumulates a higher amount of carbon. This study found that the composite footprint was increased in decomposing maize residues in both seasons, although in season one it was not significant (*P* = 0.126). The implication is that nematodes can be able to withstand greater energy carbon flow into the soil food web amended with maize residues. Finally, the predator footprint showed seasonal variations, where it was considerably higher in decomposition plots (season one) and control plots (season two). These variations could have been caused by unfavorable environmental conditions due to erratic climatic conditions. The assumption in this study was that the water content was similar across treatments but the influence of slight moisture differences cannot be ruled out.

In summary, this study demonstrates that maize residues influenced the abundance and diversity of free-living nematodes, especially the enrichment opportunist bacterivores which were lower compared with the general opportunist bacterivores and fungivores. Notably, the bacterivore, *Acrobeles,* was significantly dominant in decomposition plots in both seasons. The channel index revealed that decomposing maize residues were predominated by a fungal-mediated pathway throughout the study period. However, to ensure sustainable, active, and reliable recycling of nutrients from maize residues into the soil food web, maize residues need to be coupled with a suitable labile organic matter at the rate required by plants for their growth while considering climatic conditions.
